# Adiposity and ischemic and hemorrhagic stroke: Prospective study in women and meta-analysis

**DOI:** 10.1212/WNL.0000000000003171

**Published:** 2016-10-04

**Authors:** Mary E. Kroll, Jane Green, Valerie Beral, Cathie L.M. Sudlow, Anna Brown, Oksana Kirichek, Alison Price, TienYu Owen Yang, Gillian K. Reeves, For the Million Women Study Collaborators

**Affiliations:** From the Nuffield Department of Population Health (M.E.K., J.G., V.B., A.B., O.K., A.P., T.O.Y., G.K.R.), University of Oxford; and Centre for Clinical Brain Sciences (C.L.M.S.), University of Edinburgh, UK.

## Abstract

**Objective:**

To compare associations of body mass index (BMI) with ischemic stroke and hemorrhagic stroke risk, and to review the worldwide evidence.

**Methods:**

We recruited 1.3 million previously stroke-free UK women between 1996 and 2001 (mean age 57 years [SD 5]) and followed them by record linkage for hospital admissions and deaths. We used Cox regression to estimate adjusted relative risks for ischemic and hemorrhagic (intracerebral or subarachnoid hemorrhage) stroke in relation to BMI. We conducted a meta-analysis of published findings from prospective studies on these associations.

**Results:**

During an average follow-up of 11.7 years, there were 20,549 first strokes, of which 9,993 were specified as ischemic and 5,852 as hemorrhagic. Increased BMI was associated with an increased risk of ischemic stroke (relative risk 1.21 per 5 kg/m^2^ BMI, 95% confidence interval 1.18–1.23, *p* < 0.0001) but a decreased risk of hemorrhagic stroke (relative risk 0.89 per 5 kg/m^2^ BMI, 0.86–0.92, *p* < 0.0001). The BMI-associated trends for ischemic and hemorrhagic stroke were significantly different (heterogeneity: *p* < 0.0001) but were not significantly different for intracerebral hemorrhage (n = 2,790) and subarachnoid hemorrhage (n = 3,062) (heterogeneity: *p* = 0.5). Published data from prospective studies showed consistently greater BMI-associated relative risks for ischemic than hemorrhagic stroke with most evidence (prior to this study) coming from Asian populations.

**Conclusions:**

In UK women, higher BMI is associated with increased risk of ischemic stroke but decreased risk of hemorrhagic stroke. The totality of the available published evidence suggests that BMI-associated risks are greater for ischemic than for hemorrhagic stroke.

There is increasing recognition of adiposity as a risk factor for a wide range of common diseases in adulthood and later life,^1^ and rising levels of obesity worldwide have highlighted the importance of clarifying the relationship between adiposity and the risk of specific types of disease to inform public health policy. Stroke is a major cause of disability and death internationally^2^ and its association with body mass index (BMI) is well-established.^3^ However, this association is likely to be dominated by the association between BMI and ischemic stroke, and few prospective studies have accrued sufficient numbers of strokes of known pathologic type to estimate associations separately for ischemic and hemorrhagic stroke. Most evidence on the role of BMI in specific types of stroke comes from Asia, where stroke is more common than in Western countries and etiologies may be different.^4^ We investigated these associations in a large prospective study of UK women. To set our findings in context, we also conducted a systematic review and meta-analysis of published prospective studies relating BMI to risk of ischemic and hemorrhagic stroke.

## METHODS

### Study participants.

Between 1996 and 2001, 1.3 million middle-aged women in the United Kingdom were recruited to the Million Women Study at breast cancer screening centers in England and Scotland.^5^ Each participant returned a recruitment questionnaire on health and lifestyle characteristics, including current height and weight. In 2006–2009, direct measurements of height and weight were obtained for a subset of participants 9 years after recruitment on average.^6^ We followed the entire cohort for deaths, emigrations, and hospital admissions (as inpatients or day cases) by linkage to National Health Service (NHS) central registers and electronic hospital records. Full details of the study design, methods, survey questionnaires, and information about data sharing can be found on the study's website (www.millionwomenstudy.org).

### Exposure assessment.

Using information from the recruitment questionnaire, study participants were grouped by BMI (reported weight in kilograms divided by the square of the reported height in meters) in 5 categories: <22.5, 22.5–<25, 25–<27.5, 27.5–<30, ≥30 kg/m^2^. To allow for measurement error and changes in BMI over time, when estimating trends we scored each category as the mean measured BMI among the subset of women in that category for whom direct measurements were available: 22.2, 25.2, 27.6, 30.3, and 34.4 kg/m^2^, respectively. We defined other baseline exposures as follows: region (Scotland and the 9 extant Cancer Registry regions in England), deprivation category (a measure of socioeconomic status based on quintiles of the Townsend index,^7^ an area-based index of deprivation), self-reported strenuous exercise (“enough to cause sweating or a fast heartbeat”; none, <1, 1, 2+ times per week), alcohol intake (none, 0.5−, 3−, 7+ units per week, where 1 unit is assumed to contain 10 grams of pure alcohol), smoking (never, past, current: <15, 15+ cigarettes/d), and height (<160, 160−, 165+ centimeters). To examine the role of factors that might mediate associations of BMI with stroke,^3^ we grouped participants according to self-reported treatment for relevant conditions at recruitment: hypertension (no, yes), high blood cholesterol (no, yes), and diabetes (no, yes).

### Outcome assessment.

Women with self-reported prior stroke at recruitment, or mention of prior cerebrovascular disease (ICD-9 codes 430–438 or ICD-10 codes I60-I69) in the electronic hospital record, and those who did not report their height or weight, were excluded from all analyses. We defined the first stroke as the earliest postrecruitment hospital admission mentioning stroke, if any, or death with stroke certified as the underlying cause. Electronic hospital records were available for April 1, 1997, to March 31, 2011, in England (Hospital Episode Statistics) and January 1, 1981, to December 31, 2008, in Scotland (Scottish Morbidity Record); death and emigration records were available up to December 31, 2012. All sources provided date and causes coded to the ICD-10 for each event during the study period. Using ICD-10, we defined subarachnoid hemorrhage as I60, intracerebral hemorrhage as I61, and ischemic stroke as I63. Stroke of unspecified type (I64) includes both ischemic and hemorrhagic strokes. We used primary care data from 2 different sources to assess the numbers of strokes specified either as hemorrhagic or as ischemic in the primary care records of women whose stroke was unspecified in hospital records. Primary care data, for those women for whom this information was available, came from (1) information obtained by record linkage to the Clinical Practice Research Datalink (CPRD) (see cprd.com for further details), which is available for 8% of the cohort; and (2) that reported by primary care physicians in a postal survey requesting further information about a random subsample of 1,004 women with a hospital admission for stroke.^8^

For the small proportion of hospital stroke admissions with more than 1 recorded primary pathologic type (0.3%), we used the type recorded latest during the admission, on the assumption that diagnostic accuracy would improve during the hospital stay. A sensitivity analysis was conducted in which the endpoint was restricted to admissions with stroke as the first diagnosis listed (92% of all admissions listing stroke as a diagnosis).

### Statistical analysis.

Observation for stroke outcomes began at recruitment or the start of electronic hospital records (whichever was later) and ceased at death, emigration, or the end of electronic hospital records (whichever was earlier), with censoring at any stroke event. We estimated hazard ratios (subsequently referred to as relative risks) for stroke in each BMI category relative to a reference group (<22.5 kg/m^2^) by Cox regression, taking attained age as the underlying time variable, stratifying by region, and adjusting for potential confounders (deprivation, physical exercise, alcohol intake, smoking, and height). Women with missing values for any covariate were assigned to a separate level of that factor (deprivation <1%, exercise 3%, alcohol <1%, smoking 6%). We computed group-specific 95% confidence intervals (CIs) by estimating the variance of the log risk for each group.^9^ We also estimated log-linear trends in risk over the 5 categories of BMI, with conventional 95% CIs, scoring each category as the mean within-category measured BMI. We expressed all trends as relative risks per 5 kg/m^2^, and assessed heterogeneity between trend estimates with a 2-sided χ^2^ contrast test,^10^ using a 1% significance level (rather than 5%) to allow for repeated testing. Relative risks are presented separately after adjustment for age and region only, and after additional adjustment for deprivation, exercise, alcohol, smoking and height.

Sensitivity analyses were also conducted to assess the effect of excluding the first 5 years of follow-up (to assess potential effects of reverse causation, whereby preclinical disease may affect BMI), and of additionally excluding women with self-reported prior heart disease, thrombosis, diabetes, or cancer (not just prior stroke), and of adjustment for previous history of cancer. We also assessed the effect of excluding underweight women (BMI <18.5 kg/m^2^) from the analysis. All calculations used Stata version 13.0.^11^

We repeated the trend analyses for subgroups defined by various characteristics of the women, including age, adjustment factors, and whether or not women self-reported treatment, at baseline, for hypertension, high cholesterol, or diabetes.

To assess the plausibility of possible mechanisms for the association of BMI with specific stroke subtypes, mean apolipoprotein B/A1 ratio was calculated within categories of BMI in a subsample of women with measured lipid levels.

### Systematic review and meta-analysis.

We conducted a systematic review of prospective studies of BMI and incidence of the main pathologic types of stroke. In March 2015, we searched PubMed for relevant articles in English, using combinations of the MeSH terms stroke (both ischemic and hemorrhagic), intracerebral hemorrhage, subarachnoid hemorrhage, and body mass index. Titles and abstracts of identified articles were initially screened for relevance. Potentially relevant articles were then assessed for eligibility using the following inclusion criteria: cohort study (or analysis of pooled cohort studies using individual data) published after 1994, relating adult BMI to risk of incident stroke (fatal and nonfatal), including at least 500 strokes in total (to reduce scope for publication bias), and giving separate estimates for both hemorrhagic and ischemic stroke, adjusted for confounders (at least age, and sex or smoking where relevant) but not for potential mediators (hypertension, dyslipidemia, or diabetes). Where several models were reported, we chose the model that was adjusted for the largest number of confounders, without being adjusted for potential mediators. Where studies overlapped, we excluded the smaller study. Where necessary, trends were estimated from categorical risk estimates by generalized least squares,^12^ with group-specific BMI means estimated from means, standard deviations, or percentiles of BMI, assuming a normal distribution. Where appropriate, we combined trend estimates for subarachnoid and intracerebral hemorrhage. We expressed all trend estimates as relative risks per 5 kg/m^2^, grouped them by prespecified geographic area (Asia and Europe, North America, or Australia, because the distribution and etiologies of stroke subtypes differ between populations of Asian and European origin),^4^ and combined results by inverse-variance methods.^13^

### Standard protocol approvals, registrations, and patient consents.

The Multi-Centre Research Ethics Committee for Anglia and Oxford approved the study. Each participant gave written informed consent to follow-up through medical records.

## RESULTS

### Million Women Study.

After exclusions for prior cerebrovascular disease (1%) and missing BMI (a further 5%), 1,277,129 women were included in the analysis (table 1). Of these, 15,683 (1%) were lost to follow-up during the study period, for example, through emigration, but contributed person-years under observation until the relevant end of follow-up date. Mean age at recruitment was 56.7 years (SD 5), and mean follow-up time was 11.7 years, with 15 million person-years of follow-up included in analyses for this study. Women with higher BMI tended to have lower socioeconomic status than leaner women, and were less likely to currently smoke, drink alcohol, or use hormone therapy, but more likely to be physically inactive, and much more likely to report being on treatment for hypertension, high cholesterol, or diabetes at recruitment.

**Table 1:**
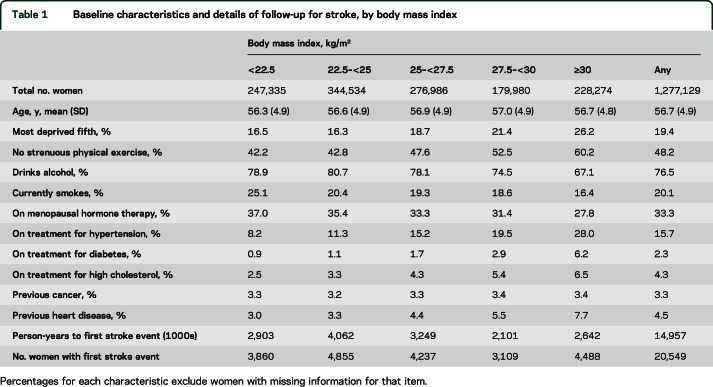
Baseline characteristics and details of follow-up for stroke, by body mass index

**Figure 1:**
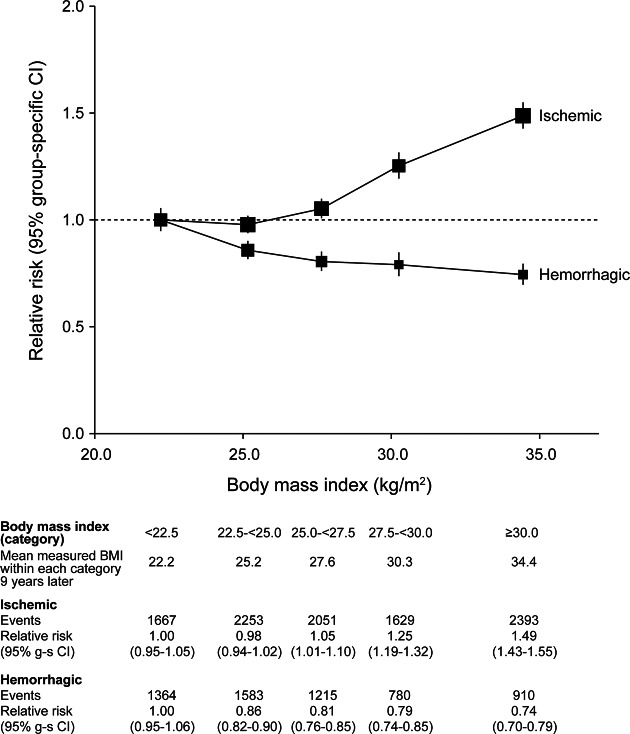
Relative risks of ischemic and hemorrhagic stroke by BMI Estimates are adjusted for age, region, deprivation, physical exercise, alcohol intake, and smoking. Relative risk for each category is plotted against the mean measured BMI in that category. The size of each square is proportional to the amount of statistical information contained. BMI = body mass index; CI = confidence interval. Published with permission from Adrian Goodill.

**Figure 2:**
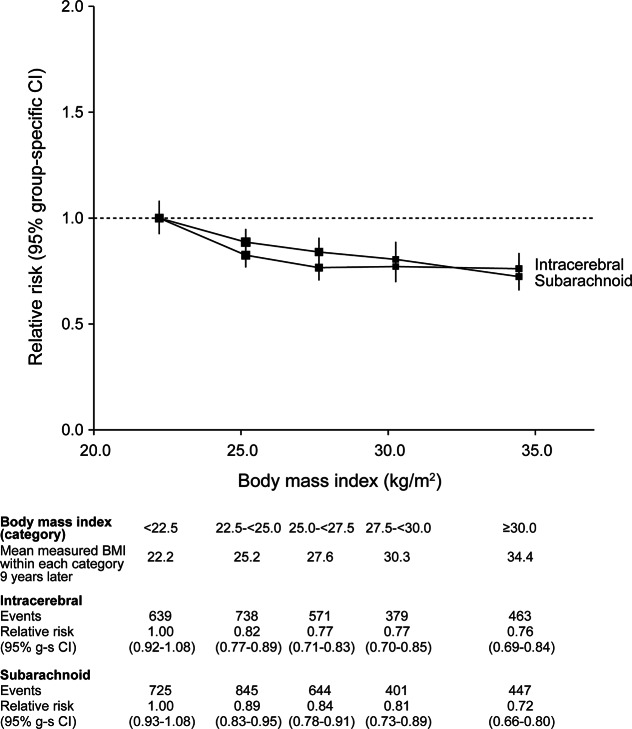
Relative risks of intracerebral and subarachnoid hemorrhage by BMI Estimates are adjusted for age, region, deprivation, physical exercise, alcohol intake, and smoking. Relative risk for each category is plotted against the mean measured BMI in that category. The size of each square is proportional to the amount of statistical information contained. BMI = body mass index; CI = confidence interval. Published with permission from Adrian Goodill.

During follow-up, there were 20,549 first strokes, of which 9,993 were coded as ischemic (cerebral infarction), 5,852 were coded as hemorrhagic (2,790 intracerebral and 3,062 subarachnoid hemorrhage), and 4,704 were of unspecified type (table 1). Increased BMI was associated with an increased risk of ischemic stroke. The relative risk of ischemic stroke per 5 kg/m^2^ increase in BMI was 1.23 (1.20–1.26) after adjustment for age and region only, and 1.21 (1.18–1.23) after additional adjustment for all 5 potential confounders listed in the Methods section. By contrast, the corresponding relative risk of hemorrhagic stroke decreased with increasing BMI with an age- and region-adjusted relative risk of 0.88 (0.85–0.91), which was relatively unaffected by adjustment for other potential confounders (relative risk 0.89, 95% CI 0.86–0.92). This difference in BMI-associated trends in risk for ischemic vs hemorrhagic stroke was highly significant (figure 1, *p* < 0.0001). There was no significant difference between the BMI-associated trends for intracerebral and subarachnoid hemorrhage (figure 2, *p* = 0.5).

The results of sensitivity analyses using only hospital admissions with stroke listed as the first diagnosis code, or excluding the first 5 years of follow-up, or excluding women with any self-reported prior ill health (not just stroke), or with adjustment for prior history of cancer, did not differ materially from the main findings (in each case, trend estimates differed by no more than 2% for both ischemic and hemorrhagic stroke). Exclusion of 12,639 (1%) women with a BMI of <18.5 kg/m^2^ had a negligible effect on the main findings (trend estimates differed by no more than 2% for both ischemic and hemorrhagic stroke).

The association between BMI and unspecified stroke (I64; n = 4,704) resembled the pattern of risk seen for any stroke except that the relative risk of unspecified stroke was slightly higher in the most obese category (figure e-1 at Neurology.org). This is likely to reflect a greater proportion of ischemic strokes among those of unspecified type.^14^ Additional information collected from primary care about women with a stroke in this cohort shows that ischemic strokes are overrepresented in the “unspecified stroke” category of hospital data. For women who were coded as having an unspecified stroke (I64) in their hospital records, but whose stroke was specified as either ischemic or hemorrhagic in their primary care records, the proportion of strokes specified as ischemic were 75% (54/72) based on linked primary care data (CPRD) and 89% (78/88) based on information provided directly by the patient's primary care physician. Both these percentages are greater than the corresponding proportion of 63% ischemic stroke (9,993/15,845), based on hospital records with specified type.

**Figure 3:**
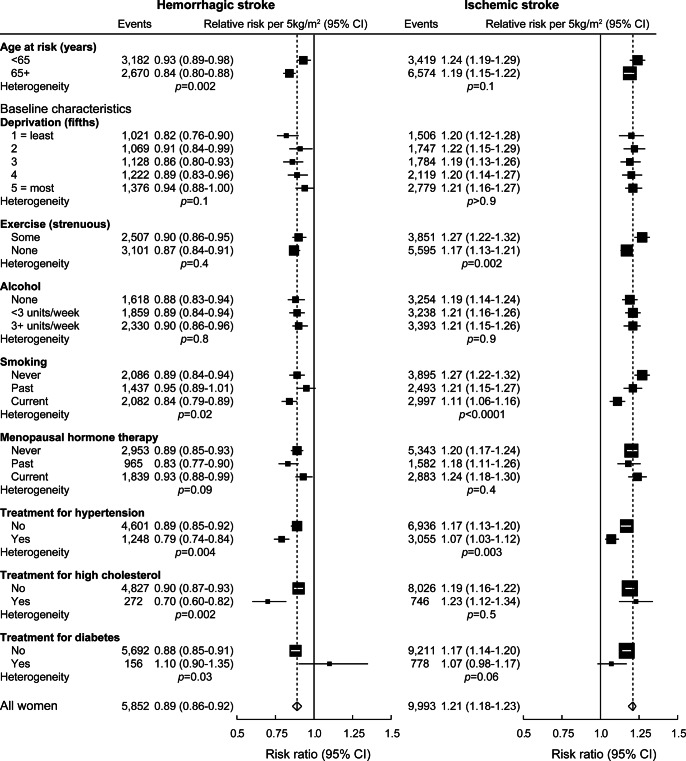
Stratified relative risks of hemorrhagic and ischemic stroke per 5 kg/m^2^ increase in body mass index Estimates are adjusted for age, region, deprivation, physical exercise, alcohol intake, and smoking, where appropriate. Person-years are classified by age at risk or baseline characteristics of participants. Dotted lines represent the respective relative risks per 5 kg/m^2^ in all women. The size of each square is proportional to the amount of statistical information contained. CI = confidence interval. Published with permission from Adrian Goodill.

There was some heterogeneity in the relative risks per 5 kg/m^2^ across subgroups of women (figure 3, *p* < 0.0001 and *p* = 0.003 for global heterogeneity among the trends for ischemic stroke and hemorrhagic stroke, respectively). For hemorrhagic stroke, the decreasing trend in relative risk with higher BMI was stronger in older women, and in those who reported at baseline being treated for hypertension or for high cholesterol. For ischemic stroke, the increasing trend in relative risk with higher BMI was weaker in physically inactive women, smokers, and those who reported at baseline being treated for hypertension. The pattern of an increasing trend in ischemic stroke and a decreasing trend in hemorrhagic stroke was evident regardless of reported treatment for high blood pressure or high cholesterol.

Among 15,739 women with information on blood lipid levels, who were not receiving treatment for high cholesterol at recruitment, mean apolipoprotein B/A1 ratio increased with increasing BMI. The mean ratios in women with BMIs (in kg/m^2^) of <25, 25–<30, and 30+ were 0.62 (0.61–0.62), 0.67 (0.66–0.67), and 0.68 (0.67–0.68), respectively.

### Systematic review.

Electronic searching retrieved 495 studies. Screening titles and abstracts eliminated 388 studies. Assessing full-text articles eliminated a further 95 studies that did not fulfil one or more specific inclusion criteria. This left 12 studies, 5 from Europe, North America, and Australia,^15–19^ and 7 from Asia^20–26^; 3 of these studies reported separate estimates for men and women (table e-1).^16,20,24^ Including our study, type-specific trend estimates were based on 52,216 stroke events in total: 30,553 from Asia and 21,656 from Europe, North America, and Australia. For Europe, North America, and Australia, previous studies contributed only 5,811 cases; our study contributed an additional 15,845 cases.

**Figure 4:**
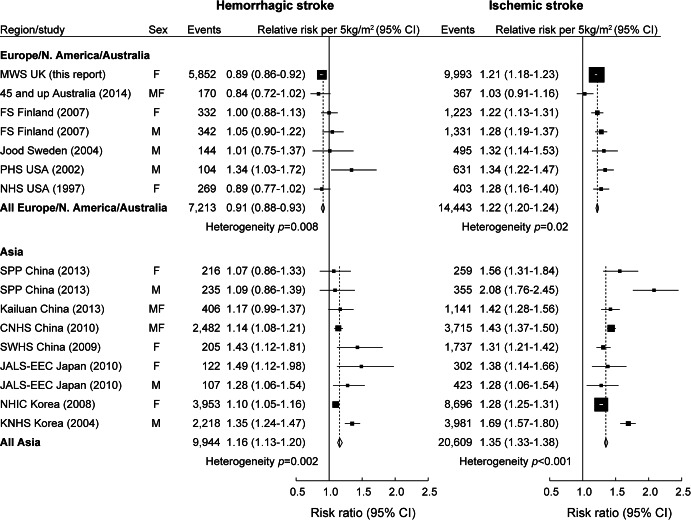
Meta-analysis of findings from this study and other published studies Relative risks of hemorrhagic and ischemic stroke per 5 kg/m^2^ increase in body mass index. Estimates are adjusted for at least age, sex, and smoking, where appropriate, but not for potential mediators (high blood pressure, high cholesterol, or diabetes). Dotted lines represent the respective relative risks per 5 kg/m^2^ in all women. The size of each square is proportional to the amount of statistical information contained. 45 and up^15^ = 45 and Up Study; CNHS^22^ = China National Hypertension Survey; FS^16^ = Finland survey; JALS-EEC^24^ = Japan Arteriosclerosis Longitudinal Study–Existing Cohorts Combine; Jood^17^ = Swedish record linkage study; Kailuan^21^ = Kailuan study; KNHS^26^ = Korean National Health System; NHIC^25^ = National Health Insurance Corporation; NHS^19^ = Nurses' Health Study; PHS^18^ = Physicians' Health Study; SPP^20^ = Stroke Prevention Project; SWHS^23^ = Shanghai Women's Health Study. CI = confidence interval. Published with permission from Adrian Goodill.

In all but 3^18,23,24^ of the 12 published studies included in the meta-analysis, the trend estimate was lower for hemorrhagic than ischemic stroke. For both stroke types, the pooled trend estimates were lower in European/North American populations than in Asian populations, and within these groups there was some heterogeneity across studies (figure 4).

## DISCUSSION

In this prospective study of 1.3 million UK women, among whom 20,549 incident strokes had occurred, increased BMI was found to be associated with an increased risk of ischemic stroke but a decreased risk of hemorrhagic stroke. The magnitude of the reduction in risk associated with increasing BMI was similar for intracerebral and subarachnoid hemorrhage.

Published findings from other prospective studies are broadly consistent with ours. The BMI-associated relative risk was greater for ischemic than for hemorrhagic stroke overall, and separately, in 9 of the 12 previously published studies that were included in the meta-analysis. Findings from 5 other potentially eligible studies that did not publish results in a suitable form for inclusion in the meta-analysis did not appear to contradict the findings of the meta-analysis.^27–31^ The difference between the trends for the 2 stroke types was greater in the European, North American and Australian studies than in the Asian studies, perhaps reflecting variation in the epidemiology of different stroke types in these different populations.^32^ As in our study, there was no evidence that the BMI association differed for subarachnoid and intracerebral hemorrhage in the 2 large Asian cohort studies that estimated separate trends for these types.^25,26^

The reasons for a difference between the BMI-associated risks of hemorrhagic and ischemic stroke are unclear. Obesity is associated with well-established risk factors for stroke in general, including hypertension, dyslipidemia, and diabetes.^33^ Hypertension increases the risk of both stroke types, but some evidence suggests that dyslipidemia and diabetes specifically increase the risk of ischemic stroke.^34^ It has also been suggested that hemorrhagic stroke risk increases with decreasing serum cholesterol levels.^35^ Although we were unable to assess directly whether differences in lipid levels could account for the observed associations between BMI and stroke subtypes, the increase in apolipoprotein B/A1 ratio with increasing BMI shown here is consistent with the hypothesis that BMI increases ischemic stroke risk, and decreases hemorrhagic stroke risk, through altered lipid levels.

We followed 1.3 million women for 11.7 years on average, using routinely collected NHS data and electronic hospital records to ascertain both fatal and nonfatal strokes with virtually no loss to follow-up. There is evidence from this^8^ and other studies^36^ that diagnoses of ischemic and hemorrhagic strokes recorded in hospital records are sufficiently reliable for epidemiologic investigation. Measurement error and changes in BMI over time were minimized by the use of measured height and weight from sampled participants 9 years after recruitment to score BMI categories for trend estimation.

Comparisons with primary care data, and published data on the relative frequency of pathologic types of stroke in populations of European origin,^14^ suggest that relatively few women coded as having had an unspecified stroke in the hospital data had had a hemorrhagic stroke. Thus incomplete information on stroke types in hospital data is unlikely to have materially biased the association of increased BMI with decreased risk of hemorrhagic stroke observed here. Nor are reverse causation or confounding likely to explain the results, since trend estimates changed very little after excluding the first 5 years of follow-up, and the direction of the trends differed between the 2 types of stroke. Our analyses are based on strokes occurring in women over the age of 50 that resulted in hospital admission or death. Since hemorrhagic strokes are more likely than ischemic strokes to result in hospital admission or death,^37,38^ this is likely to have led to an overrepresentation of hemorrhagic strokes in this study compared with other studies where stroke outcome was ascertained differently. This should not, however, bias our estimates of the association between BMI and specific types of stroke and, while we cannot necessarily assume that the findings observed here apply equally to strokes managed without admission to hospital, our findings are unlikely to have been materially affected by omission of this subgroup of stroke cases.

The main finding from this study is that greater adiposity is associated with an increase in ischemic stroke, but not necessarily hemorrhagic stroke. Our results suggest qualitatively different BMI-associated risks for these 2 pathologic types of stroke in UK women, and a decreased risk of both subarachnoid and intracerebral hemorrhage with higher BMI.

## Supplementary Material

Data Supplement

Coinvestigators

Accompanying Editorial

## GLOSSARY

BMI: body mass index
CI: confidence interval
CPRD: Clinical Practice Research Datalink
*ICD-9*: *International Classification of Diseases–9*
*ICD-10*: *International Classification of Diseases–10*
NHS: National Health Service

## References

[1] Reeves GK, Balkwill A, Cairns BJ, Green J, Beral V; Million Women Study Collaborators. Hospital admissions in relation to body mass index in UK women: a prospective cohort study. BMC Med 2014;12:45. 24629170 10.1186/1741-7015-12-45 PMC4003825

[2] Krishnamurthi RV, Feigin VL, Forouzanfar MH, et al. Global and regional burden of first-ever ischaemic and haemorrhagic stroke during 1990-2010: findings from the Global Burden of Disease Study 2010. Lancet Glob Health 2013;1:e259–e281. 25104492 10.1016/S2214-109X(13)70089-5 PMC4181351

[3] Goldstein LB, Bushnell CD, Adams RJ, et al. Guidelines for the primary prevention of stroke: a guideline for healthcare professionals from the American Heart Association/American Stroke Association. Stroke 2011;42:517–584. 21127304 10.1161/STR.0b013e3181fcb238

[4] Tsai CF, Thomas B, Sudlow CL. Epidemiology of stroke and its subtypes in Chinese vs white populations: a systematic review. Neurology 2013;81:264–272. 23858408 10.1212/WNL.0b013e31829bfde3 PMC3770160

[5] Reeves GK, Pirie K, Beral V, Green J, Spencer E, Bull D. Cancer incidence and mortality in relation to body mass index in the Million Women Study: cohort study. BMJ 2007;335:1134–1139. 17986716 10.1136/bmj.39367.495995.AE PMC2099519

[6] Wright FL, Green J, Reeves G, Beral V, Cairns BJ; Million Women Study collaborators. Validity over time of self-reported anthropometric variables during follow-up of a large cohort of UK women. BMC Med Res Methodol 2015;15:81. 26450616 10.1186/s12874-015-0075-1 PMC4599695

[7] Townsend PP, Beattie A. Health and Deprivation: Inequality and the North. London: Croom Helm; 1988.

[8] Wright FL, Green J, Canoy D, et al. Vascular disease in women: comparison of diagnoses in hospital episode statistics and general practice records in England. BMC Med Res Methodol 2012;12:161. 23110714 10.1186/1471-2288-12-161 PMC3514155

[9] Beral V, Hermon C, Peto R, et al. Ovarian cancer and body size: individual participant meta-analysis including 25,157 women with ovarian cancer from 47 epidemiological studies. Plos Med 2012;9:e1001200. 22606070 10.1371/journal.pmed.1001200 PMC3317899

[10] Smith-Warner SA, Spiegelman D, Ritz J, et al. Methods for pooling results of epidemiologic studies: the pooling project of prospective studies of diet and cancer. Am J Epidemiol 2006;163:1053–1064. 16624970 10.1093/aje/kwj127

[11] Stata Statistical Software. Release 13. College Station, TX: StataCorp LP; 2013.

[12] Greenland S, Longnecker MP. Methods for trend estimation from summarized dose-response data, with applications to metaanalysis. Am J Epidemiol 1992;135:1301–1309. 1626547 10.1093/oxfordjournals.aje.a116237

[13] Higgins JP, Thompson SG. Quantifying heterogeneity in a meta-analysis. Stat Med 2002;21:1539–1558. 12111919 10.1002/sim.1186

[14] Feigin VL, Lawes CM, Bennett DA, Barker-Collo SL, Parag V. Worldwide stroke incidence and early case fatality reported in 56 population-based studies: a systematic review. Lancet Neurol 2009;8:355–369. 19233729 10.1016/S1474-4422(09)70025-0

[15] Joshy G, Korda RJ, Attia J, et al. Body mass index and incident hospitalisation for cardiovascular disease in 158,546 participants from the 45 and UP Study. Int J Obes 2014;38:848–856. 10.1038/ijo.2013.192 PMC4052432 24149770

[16] Hu G, Tuomilehto J, Silventoinen K, Sarti C, Mannisto S, Jousilahti P. Body mass index, waist circumference, and waist-hip ratio on the risk of total and type-specific stroke. Arch Intern Med 2007;167:1420–1427. 17620537 10.1001/archinte.167.13.1420

[17] Jood K, Jern C, Wilhelmsen L, Rosengren A. Body mass index in mid-life is associated with a first stroke in men: a prospective population study over 28 years. Stroke 2004;35:2764–2769. 15514172 10.1161/01.STR.0000147715.58886.ad

[18] Kurth T, Gaziano JM, Berger K, et al. Body mass index and the risk of stroke in men. Arch Intern Med 2002;162:2557–2562. 12456227 10.1001/archinte.162.22.2557

[19] Rexrode KM, Hennekens CH, Willett WC, et al. A prospective study of body mass index, weight change, and risk of stroke in women. JAMA 1997;277:1539–1545. 9153368 10.1001/jama.1997.03540430051032

[20] Wang C, Liu Y, Yang Q, et al. Body mass index and risk of total and type-specific stroke in Chinese adults: results from a longitudinal study in China. Int J Stroke 2013;8:245–250. 23039874 10.1111/j.1747-4949.2012.00830.x

[21] Wang A, Wu J, Zhou Y, et al. Measures of adiposity and risk of stroke in China: a result from the Kailuan Study. PLoS One 2013;8:e61665. 23613897 10.1371/journal.pone.0061665 PMC3629147

[22] Bazzano LA, Gu D, Whelton MR, et al. Body mass index and risk of stroke among Chinese men and women. Ann Neurol 2010;67:11–20. 20186847 10.1002/ana.21950 PMC4371851

[23] Zhang X, Shu XO, Gao YT, Yang G, Li H, Zheng W. General and abdominal adiposity and risk of stroke in Chinese women. Stroke 2009;40:1098–1104. 19211490 10.1161/STROKEAHA.108.539692 PMC2663595

[24] Yatsuya H, Toyoshima H, Yamagishi K, et al. Body mass index and risk of stroke and myocardial infarction in a relatively lean population: meta-analysis of 16 Japanese cohorts using individual data. Circ Cardiovasc Qual Outcomes 2010;3:498–505. 20699444 10.1161/CIRCOUTCOMES.109.908517

[25] Park JW, Lee SY, Kim SY, Choe H, Jee SH. BMI and stroke risk in Korean women. Obesity 2008;16:396–401. 18239650 10.1038/oby.2007.67

[26] Song YM, Sung J, Smith GD, Ebrahim S. Body mass index and ischemic and hemorrhagic stroke: a prospective study in Korean men. Stroke 2004;35:831–836. 15001798 10.1161/01.STR.0000119386.22691.1C

[27] The Asia Pacific Cohort Studies Collaboration. Impact of cigarette smoking on the relationship between body mass index and coronary heart disease: a pooled analysis of 3264 stroke and 2706 CHD events in 378579 individuals in the Asia Pacific region. BMC Public Health 2009;9:294. 19678933 10.1186/1471-2458-9-294 PMC2734855

[28] Ni Mhurchu C, Rodgers A, Pan WH, Gu DF, Woodward M; Asia Pacific Cohort Studies Collaboration. Body mass index and cardiovascular disease in the Asia-Pacific Region: an overview of 33 cohorts involving 310,000 participants. Int J Epidemiol 2004;33:751–758. 15105409 10.1093/ije/dyh163

[29] Saito I, Iso H, Kokubo Y, Inoue M, Tsugane S. Body mass index, weight change and risk of stroke and stroke subtypes: the Japan Public Health Center-based prospective (JPHC) study. Int J Obes 2011;35:283–291. 10.1038/ijo.2010.131 20603628

[30] Hyun KK, Huxley RR, Arima H, et al. A comparative analysis of risk factors and stroke risk for Asian and non-Asian men: the Asia Pacific Cohort Studies Collaboration. Int J Stroke 2013;8:606–611. 24148094 10.1111/ijs.12166

[31] Larsson SC, Akesson A, Wolk A. Healthy diet and lifestyle and risk of stroke in a prospective cohort of women. Neurology 2014;83:1699–1704. 25298305 10.1212/WNL.0000000000000954 PMC4239835

[32] Flaherty ML, Woo D, Broderick JP. The Epidemiology of Intracerebral Hemorrhage. In: Carhuapoma JR, Mayer SA, Hanley DF, eds. Intracerebral Hemorrhage. Cambridge: Cambridge University Press; 2010:1–10.

[33] Global Burden of Metabolic Risk Factors for Chronic Diseases Collaboration (BMI Mediated Effects); Lu Y, Hajifathalian K, Ezzati M, Woodward M, Rimm EB, Danaei G. Metabolic mediators of the effects of body-mass index, overweight, and obesity on coronary heart disease and stroke: a pooled analysis of 97 prospective cohorts with 1·8 million participants. Lancet 2014;383:970–983. 24269108 10.1016/S0140-6736(13)61836-X PMC3959199

[34] O'Donnell MJ, Xavier D, Liu L, et al. Risk factors for ischaemic and intracerebral haemorrhagic stroke in 22 countries (the INTERSTROKE study): a case-control study. Lancet 2010;376:112–123. 20561675 10.1016/S0140-6736(10)60834-3

[35] Wang X, Dong Y, Qi X, Huang C, Hou L. Cholesterol levels and risk of hemorrhagic stroke. A systematic review and meta-analysis. Stroke 2013;44:1833–1839. 23704101 10.1161/STROKEAHA.113.001326

[36] Woodfield RM, Grant I, Sudlow CL; UK Biobank Stroke Outcomes Group; UK Biobank Follow-Up; Outcomes Working Group. Accuracy of electronic health record data for identifying stroke cases in large-scale epidemiological studies: a systematic review from the UK Biobank stroke outcomes group. PLoS One 2015;10:e0140533. 26496350 10.1371/journal.pone.0140533 PMC4619732

[37] Andersen KK, Olsen TS, Dehlendorff C, Kammersgaard LP. Hemorrhagic and ischaemic strokes compared: stoke severity, mortality, and risk factors. Stroke 2009;40:2068–2072. 19359645 10.1161/STROKEAHA.108.540112

[38] Bamford J, Dennis M, Sandercock P, Burn J, Warlow C. The frequency, causes and timing of death within 30 days of a first stroke: the Oxfordshire Community Stroke Project. J Neurol Neurosurg Psychiatry 1990;53:824–829. 2266360 10.1136/jnnp.53.10.824 PMC488240

