# Supplementary material for: Adiposity and ischemic and hemorrhagic stroke: Prospective study in women and meta-analysis
Source: Neurology. 2016 Oct 4;87(14):1473–81. doi: 10.1212/WNL.0000000000003171 (PMC5075975; doi:10.1212/WNL.0000000000003171)
Supplement: Data Supplement [file supp_WNL.0000000000003171_eFigure1_revised.pdf]

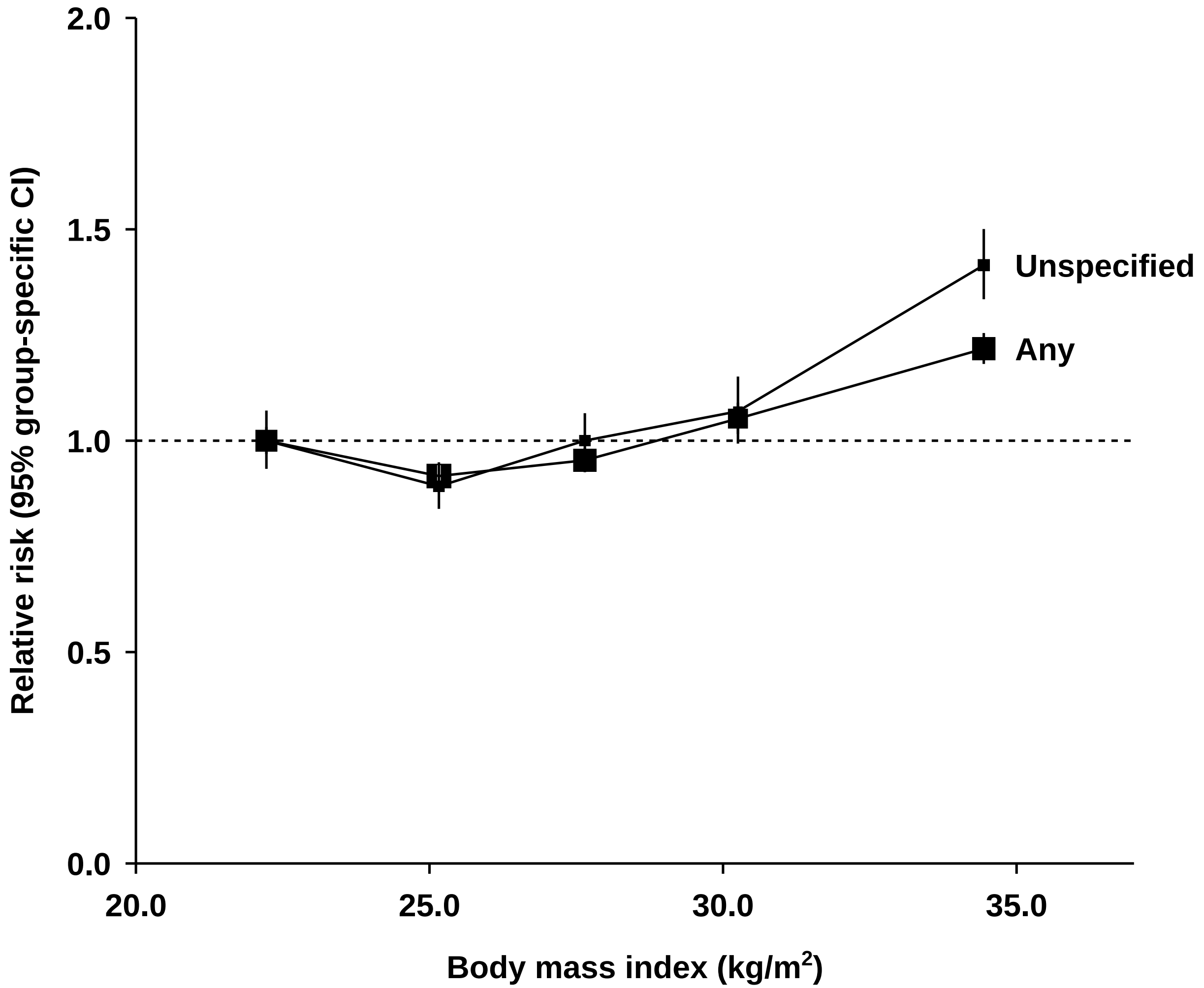

Body mass index (category)	<22.5	22.5-<25.0	25.0-<27.5	27.5-<30.0	≥30.0
Mean measured BMI within each category 9 years later	22.2	25.2	27.6	30.3	34.4
**Unspecified**					
Events	829	1019	971	700	1185
Relative risk	1.00	0.89	1.00	1.07	1.42
(95% g-s CI)	(0.93-1.07)	(0.84-0.95)	(0.94-1.07)	(0.99-1.15)	(1.33-1.50)
**Any**					
Events	3860	4855	4237	3109	4488
Relative risk	1.00	0.92	0.95	1.05	1.22
(95% g-s CI)	(0.97-1.03)	(0.89-0.94)	(0.93-0.98)	(1.02-1.09)	(1.18-1.25)
