# Supplementary material for: Adiposity and ischemic and hemorrhagic stroke: Prospective study in women and meta-analysis
Source: Neurology. 2016 Oct 4;87(14):1473–81. doi: 10.1212/WNL.0000000000003171 (PMC5075975; doi:10.1212/WNL.0000000000003171)
Supplement: Data Supplement [file supp_WNL.0000000000003171_table_e-1.docx]

Table e-1. Published findings for meta-analysis. Non-overlapping cohort studies that were published in English after 1994, related incident stroke to adult body mass index, included at least 500 cases of stroke, reported (or provided sufficient information to estimate) separate trends for both ischaemic and haemorrhagic stroke, and adjusted for confounders but not for potential mediators (blood pressure, blood lipids or diabetes).

|  | **Europe, North America, Australia** | | | | | | **Asia** | | | | | | | | |
| --- | --- | --- | --- | --- | --- | --- | --- | --- | --- | --- | --- | --- | --- | --- | --- |
| **Study** | **45 & up** | **FS** | **FS** | **Jood** | **PHS** | **NHS** | **SPP** | **SPP** | **Kailuan** | **CHNS** | **SWHS** | **JALS-EEC** | **JALS-EEC** | **NHIC** | **KNHS** |
| **Setting** | **Australia** | **Finland** | **Finland** | **Sweden** | **USA** | **USA** | **China** | **China** | **China** | **China** | **China** | **Japan** | **Japan** | **Korea** | **Korea** |
| **Year published** | 2014 | 2007 | 2007 | 2004 | 2002 | 1997 | 2013 | 2013 | 2013 | 2010 | 2009 | 2010 | 2010 | 2008 | 2004 |
| **Participants** | 158,546 | 26,029 | 23,967 | 7495 | 21,414 | 116,759 | 14,047 | 12,560 | 94,744 | 154,736 | 67,083 | 25,475 | 19,760 | 439,582 | 234,863 |
| **Baseline characteristics** |  |  |  |  |  |  |  |  |  |  |  |  |  |  |  |
| Female | 56% | 100% | 0% | 0% | 0% | 100% | 100% | 0% | 20% | 51% | 100% | 100% | 0% | 100% | 0% |
| **Age in years, mean** | 57.8 | 44.4 | 44.0 | 51.6 | 53.1 | a | 51.4 | 54.0 | 51.2 | 55.9 | 51.3 | 56.5 | 55.4 | 49.2 | 47.1 |
| **Person-years, mean** | 3.4 | 20.2 | 18.8 | 28 | 12.5 | 14.7 | 11 b | 11 b | 4 c | 8.3 | 7.3 | d | d | e | 9.6 |
| **Events** |  |  |  |  |  |  |  |  |  |  |  |  |  |  |  |
| Ischaemic | 367 | 1223 | 1331 | 495 | 631 | 403 | 259 | 355 | 1141 | 3715 | 1737 | 302 | 423 | 8696 f | 3981 |
| haemorrhagic | 170 f | 332 | 342 | 144 | 104 | 269 | 216 | 235 | 406 | 2482 | 205 | 122 | 107 | 3953 f | 2218 g |
| Any stroke | 1447 | 1555 | 1673 | 873 | 747 | 866 | 507 | 592 | 1547 | 7489 | 2403 | 537 | 576 | 16535 h | 7444 |
| **Covariates** |  |  |  |  |  |  |  |  |  |  |  |  |  |  |  |
| Age | x | x | x | x | x | x | x | x | x | x | x | x |  | x | x |
| Smoking | x | x | x | x | x | x | x | x | x | x | x | x |  | c | x |
| Alcohol | x | x | x |  | x |  | x | x | x | x | x | x |  | x | x |
| Exercise |  | x | x | x | x |  |  |  |  | x | x |  |  | x | x |
| Education | x | x | x |  |  |  | x | x | x | x | x |  |  |  |  |
| Family history |  | x | x | x | x |  |  |  |  |  |  |  |  |  |  |
| Time period |  | x | x |  |  | x |  |  |  |  |  |  |  |  |  |
| Others | h |  |  | i | j | k |  |  | l | m | n |  |  |  | o |

^a^ range 30-55 years; ^b^ follow-up period 11 years; ^c^ follow-up period 4 years; ^d^337,287 person-years of follow-up; ^e^follow-up period 13 years; ^f^ separate estimates for subarachnoid and intercerebral haemorrhage; ^g^ never-smokers only (94% of participants); ^h^ sex, income, region of residence, health insurance; ^j^ angina, randomisaation; ^k^ occupational class, psychological stress; ^j^hormone therapy, menopause, oral contraception,; ^l^income, marital status, physical activity; ^m^province, region, sex, urban; ^n^ aspirin family income, occupation, oral contraceptives, hormone therapy, aspirin, intake of saturated fat, vegetables, fruits and sodium ; ^o^income.

45 and up^1^ 45 and up Study; FS^2^ Finland Survey; Jood^3^ Swedish record linkage study; PHS^4^ Physicians’ Health Study; NHS^5^ Nurses’ Health Study; SPP^6^ Stroke Prevention Project; Kailuan^7^  Kailuan Study; CNHS^8^  China National Hypertension Survey; SWHS^9^ Shanghai Women’s Health Study; JALS-EEC^10^ Japan Arteriosclerosis Longitudinal Study-Existing Cohorts Combine; NHIC^11^ National Health Insurance Corporation; KNHS^12^ Korean National Health System.

References:

1. Joshy G, Korda RJ, Attia J, Liu B, et al. Body mass index and incident hospitalisation for cardiovascular disease in 158,546 participants from the 45 and UP Study. Int J Cancer 2014; 38: 848-856.

2. Hu G, Tuomilehto J, Silventoinen K, Sarti C, Mannisto S, Jousilahti P. Body mass index, waist circumference, and waist-hip ratio on the risk of total and type-specific stroke. Arch Intern Med 2007;167:1420-1427.

3. Jood K, Jern C, Wilhelmsen L, Rosengren A. Body mass index in mid-life is associated with a first stroke in men: a prospective population study over 28 years. Stroke 2004;35:2764-2769.

4. Kurth T, Gaziano JM, Berger K, et al. Body mass index and the risk of stroke in men. Arch Intern Med 2002;162:2557-2562.

5. Rexrode KM, Hennekens CH, Willett WC, et al. A prospective study of body mass index, weight change, and risk of stroke in women. JAMA 1997;277:1539-1545.

6. Wang C, Liu Y, Yang Q, Dai x, et al. Body mass index and risk of total and type-specific stroke in Chinese adults: results from a longitudinal study in China. International J Stroke 2013; 8: 245-250.

7. Wang A, Wu J, Zhou Y, Guo X, et al. Measures of adiposity and risk of stroke in China: a result from the Kailuan Study. PLoS One 2013; 8: e61665.

8. Bazzano LA, Gu D, Whelton MR, et al. Body mass index and risk of stroke among Chinese men and women. Ann Neurol 2010;67:11-20. 21.

9. Zhang X, Shu XO, Gao YT, Yang G, Li H, Zheng W. General and abdominal adiposity and risk of stroke in Chinese women. Stroke 2009;40:1098-1104.

10. Yatsuya H, Toyoshima H, Yamagishi K, et al. Body mass index and risk of stroke and myocardial infarction in a relatively lean population: meta-analysis of 16 Japanese cohorts using individual data. Circ Cardiovasc Qual Outcomes 2010;3:498-505.

11. Park JW, Lee S-Y, Kim SY, Choe H, Jee SH. BMI and stroke risk in Korean women. Obesity 2008;16:396-401.

12. Song YM, Sung J, Smith GD, Ebrahim S. Body mass index and ischemic and hemorrhagic stroke: a prospective study in Korean men. Stroke 2004;35:831-836.
