# Supplementary material for: Adiposity and ischemic and hemorrhagic stroke: Prospective study in women and meta-analysis
Source: Neurology. 2016 Oct 4;87(14):1473–81. doi: 10.1212/WNL.0000000000003171 (PMC5075975; doi:10.1212/WNL.0000000000003171)
Supplement: Coinvestigators [file supp_87_14_1473_v2_index.html]

Adiposity and ischemic and hemorrhagic stroke — Coinvestigators 

# Adiposity and ischemic and hemorrhagic stroke

## Coinvestigators

**Neurology® data supplements are not copyedited before publication. Published editorials and translations have been copyedited.  
 © 2016 American Academy of Neurology.  
  
 Files in this Data Supplement:**

- Coinvestigators - Microsoft Word file
