# Supplementary material for: Adiposity and ischemic and hemorrhagic stroke: Prospective study in women and meta-analysis
Source: Neurology. 2016 Oct 4;87(14):1473–81. doi: 10.1212/WNL.0000000000003171 (PMC5075975; doi:10.1212/WNL.0000000000003171)
Supplement: Coinvestigators [file supp_WNL.0000000000003171_Coinvestigators.docx]

**Million Women Study contributors and collaborating centres**

The Million Women Study is co-ordinated from the Cancer Epidemiology Unit in the Nuffield Department of Public Health, Oxford University, UK.

**Advisory Committee members:** Emily Banks (Australian National University, PhD), Valerie Beral (University of Oxford, FRS), Lucy Carpenter (Nuffield College, Oxford, PhD), Carol Dezateux (University College, London, MBBS), Jane Green (University of Oxford, DPhil), Julietta Patnick (University of Oxford, BA), Richard Peto (University of Oxford, FRS), Cathie Sudlow (University of Edinburgh, DPhil).

**Million Women Study Co-ordinating Centre staff:** Hayley Abbiss, Simon Abbott, Rupert Alison, Naomi Allen, Miranda Armstrong, Krys Baker, Angela Balkwill, Emily Banks, Isobel Barnes, Valerie Beral, Judith Black, Roger Blanks, Kathryn Bradbury, Anna Brown, Benjamin Cairns, Karen Canfell, Dexter Canoy, Andrew Chadwick, Barbara Crossley, Francesca Crowe, Dave Ewart, Sarah Ewart, Lee Fletcher, Sarah Floud, Toral Gathani, Laura Gerrard, Adrian Goodill, Jane Green, Lynden Guiver, Michal Hozak, Isobel Lingard, Sau Wan Kan, Oksana Kirichek, Nicky Langston, Bette Liu, Kath Moser, Kirstin Pirie, Gillian Reeves, Keith Shaw, Emma Sherman, Helena Strange, Sian Sweetland, Sarah Tipper, Ruth Travis, Lyndsey Trickett, Lucy Wright, Owen Yang, Heather Young.

**The following NHS Breast Screening Centres took part in the recruitment and breast screening follow up for the Million Women Study:** Avon, Aylesbury, Barnsley, Basingstoke, Bedfordshire and Hertfordshire, Cambridge and Huntingdon, Chelmsford and Colchester, Chester, Cornwall, Crewe, Cumbria, Doncaster, Dorset, East Berkshire, East Cheshire, East Devon, East of Scotland, East Suffolk, East Sussex, Gateshead, Gloucestershire, Great Yarmouth, Hereford and Worcester, Kent, Kings Lynn, Leicestershire, Liverpool, Manchester, Milton Keynes, Newcastle, North Birmingham, North East Scotland, North Lancashire, North Middlesex, North Nottingham, North of Scotland, North Tees, North Yorkshire, Nottingham, Oxford, Portsmouth, Rotherham, Sheffield, Shropshire, Somerset, South Birmingham, South East Scotland, South East Staffordshire, South Derbyshire, South Essex, South Lancashire, South West Scotland, Surrey, Warrington Halton St Helens and Knowsley, Warwickshire Solihull and Coventry, West Berkshire, West Devon, West London, West Suffolk, West Sussex, Wiltshire, Winchester, Wirral, Wycombe.
