# Supplementary material for: Adiposity and ischemic and hemorrhagic stroke: Prospective study in women and meta-analysis
Source: Neurology. 2016 Oct 4;87(14):1473–81. doi: 10.1212/WNL.0000000000003171 (PMC5075975; doi:10.1212/WNL.0000000000003171)
Supplement: Accompanying Editorial [file supp_WNL.0000000000003171_1432.pdf]

EDITORIAL

Body mass index and stroke in UK women

"Obesity paradox" revisited

Kathryn Rexrode, MD, MPH
Tatjana Rundek, MD, PhD

Correspondence to Dr. Rexrode: krexrode@partners.org

Neurology® 2016;87:1432–1433

Obesity is a major risk factor for stroke. However, several studies have reported a protective effect of obesity on stroke, particularly after a prior cardiovascular event, a phenomenon described as the "obesity paradox" or "reverse epidemiology."[1] No convincing biological evidence explains this paradox. The evidence that increased body mass index (BMI) increases risk for ischemic stroke in Western and Asian populations is strong, but the relationship with hemorrhagic stroke remains less established.

In this issue of *Neurology*®, Kroll et al.[2] analyze the association between BMI and stroke in UK women, evaluating potential differences by type of stroke: total, ischemic, and hemorrhagic, and perform a meta-analysis of BMI and stroke from Western and Asian populations.

The primary analysis was conducted in the Million Women Study, a study of 1.3 million middle-aged women in the United Kingdom recruited between 1997 and 2001, who reported height, weight, and additional information by questionnaire. Among women without a report of prior cerebrovascular disease, more than 20,000 stroke hospitalizations and deaths occurred during an average follow-up of more than 11 years. Higher BMI was directly associated with increased risk of ischemic stroke, with a 21% increased risk of ischemic stroke for each 5 kg/m^2 increase in BMI. In contrast, higher BMI was associated with a 12% reduction in hemorrhagic stroke risk for each 5 kg/m^2 increase. Overall, risk for any stroke was increased among women with a BMI of 26.5 kg/m^2 or higher, with a 22% increased risk among those with BMI of 30 or higher.[2]

Data have consistently demonstrated the relationship between obesity and coronary heart disease,[3] but the relationship with stroke has been less clear, perhaps due in part to the divergent association by type of stroke,[4] leading to different estimates based on the proportion of hemorrhagic and ischemic strokes. The current report, based on a large number of stroke events, confirms different associations for BMI with ischemic and hemorrhagic stroke, at least in Western women.

The authors also performed a meta-analysis of existing data on the association of BMI with hemorrhagic and ischemic stroke that duplicated the findings for ischemic stroke in Asian populations, but demonstrated a different association for BMI and hemorrhagic stroke in Asian populations. For ischemic stroke, a 5 kg/m^2 increase in BMI was associated with a 22% increased risk in European/North American/Australian cohorts and a 35% increased risk among the Asian populations. However, for hemorrhagic stroke, a 9% reduction in risk was estimated for each 5 kg/m^2 in the European/North American/Australian cohorts, compared with a 16% increased risk of hemorrhagic stroke per 5 kg/m^2 among the Asian cohorts.[2] Thus, the relationship with BMI and ischemic stroke seems consistent across different race ethnicity groups, although Asians have higher risk, associated with a 5 kg/m^2 increase, than non-Asians have. The markedly stronger association between BMI and ischemic stroke in Asian populations is consistent with the observation that negative metabolic changes, such as dyslipidemia and dysglycemia, are observed at lower levels of excess body weight in Asians than non-Asians.[5]

In contrast, the results for BMI and hemorrhagic stroke diverge, with non-Asian populations experiencing a 9% risk reduction for every 5 kg/m^2 increase in BMI, but Asian populations having a 16% increase in hemorrhagic stroke for the same increase in weight. The meta-analyses did not analyze separately by sex, and more than half of the events for both stroke types were contributed by the current Million Women Study.

Why is increasing adiposity associated with stroke? Many major risk factors for ischemic stroke, hypertension, diabetes, and elevated cholesterol, all increase with increasing adiposity. Mendelian randomization analyses have shown that a genetic risk score predicting BMI also is associated with an increased risk of ischemic stroke.[6] Like adiposity, lipids have a different relationship for ischemic and hemorrhagic stroke. Increasing adiposity is associated with higher total cholesterol and low-density lipoprotein (LDL) cholesterol, as well as apolipoprotein B/A1 ratio.[2] However, total cholesterol and LDL cholesterol are inversely related to risk of hemorrhagic stroke,[7] while the total cholesterol/high-density lipoprotein ratio has been consistently related to ischemic stroke.[8] The degree to which total or LDL

See page 1473

From the Division of Preventive Medicine (K.R.), Department of Medicine, Brigham and Women's Hospital, Boston, MA; and Department of Neurology (T.R.), University of Miami Miller School of Medicine, FL.

Go to Neurology.org for full disclosures. Funding information and disclosures deemed relevant by the authors, if any, are provided at the end of the editorial.

© 2016 American Academy of Neurology

© 2016 American Academy of Neurology. Unauthorized reproduction of this article is prohibited.

cholesterol might mediate the BMI relationship with hemorrhagic stroke could not be examined in the current study but would be of interest. Additional research on the mechanisms by which low BMI affects hemorrhagic stroke risk is needed.

The stronger association of BMI with both hemorrhagic and ischemic stroke in Asian populations presents a striking finding, given the rising prevalence for obesity worldwide. Asian populations demonstrate cardiometabolic effects at even lower BMI thresholds than in European populations.[5] Given the direct relationship with both ischemic and hemorrhagic stroke in Asian populations, they will endure even greater effects of the scourge of rising obesity.

Importantly, in every BMI category, higher BMI was associated with increased risk of total stroke, and the number of ischemic strokes exceeded hemorrhagic stroke in every category. Thus, higher BMI did not protect or reduce risk of total stroke. Moreover, obesity is an important risk stroke factor for all ages and even more alarming for young adults.[9] The Million Women Study was designed as a breast cancer screening study and therefore is limited for the ascertainment of detailed stroke risk factors. BMI is a crude measure of obesity and does not take into account fitness, nutritional status, or body fat distribution. The discussion over the existence of the obesity paradox, especially if defined by BMI, should not underestimate the importance of obesity as a risk factor for stroke, cardiovascular disease, and metabolic diseases, which requires far-reaching management and prevention.

STUDY FUNDING

No targeted funding reported.

DISCLOSURE

The authors report no disclosures relevant to the manuscript. Go to Neurology.org for full disclosures.

REFERENCES

1. Katsnelson M, Rundek T. Obesity paradox and stroke: noticing the (fat) man behind the curtain. Stroke 2011; 42:3331–3332.
2. Kroll ME, Green J, Beral V, et al. Adiposity and ischemic and hemorrhagic stroke: prospective study in women and meta-analysis. Neurology 2016;87:1473–1481.
3. Mongraw-Chaffin ML, Peters SA, Huxley RR, Woodward M. The sex-specific association between BMI and coronary heart disease: a systematic review and meta-analysis of 95 cohorts with 1.2 million participants. Lancet 2015;3:437–449.
4. Rexrode KM, Hennekens CH, Willett WC, et al. A prospective study of body mass index, weight change, and risk of stroke in women. JAMA 1997;277:1539–1545.
5. Prasad DS, Kabir Z, Dash AK, Das BC. Effect of obesity on cardiometabolic risk factors in Asian Indians. J Cardiovasc Dis Res 2013;4:116–122.
6. Hagg S, Fall T, Ploner A, et al. Adiposity as a cause of cardiovascular disease: a Mendelian randomization study. Int J Epidemiol 2015;44:578–586.
7. Wang X, Dong Y, Qi X, Huang C, Hou L. Cholesterol levels and risk of hemorrhagic stroke: a systematic review and meta-analysis. Stroke 2013;44:1833–1839.
8. Zhang Y, Tuomilehto J, Jousilahti P, Wang Y, Antikainen R, Hu G. Total and high-density lipoprotein cholesterol and stroke risk. Stroke 2012;43:1768–1774.
9. Mitchell AB, Cole JW, McArdle PF, et al. Obesity increases risk of ischemic stroke in young adults. Stroke 2015;46: 1690–1692.

© 2016 American Academy of Neurology. Unauthorized reproduction of this article is prohibited.
